# Cross-Cultural Adaptation and Validation of the Serbian Version of the Brief Assessment of Cognition in Schizophrenia Scale

**DOI:** 10.3390/ijerph20043699

**Published:** 2023-02-19

**Authors:** Sanja Totic Poznanovic, Milos Markovic, Milena Stasevic, Ivana Stasevic Karlicic, Milena Tomanic

**Affiliations:** 1 Faculty of Medicine, University of Belgrade, 11000 Belgrade, Serbia; 2 Clinic for Psychiatry, University Clinical Centre of Serbia, 11000 Belgrade, Serbia; 3 Clinic for Mental Disorders “Dr Laza Lazarevic”, 11000 Belgrade, Serbia; 4 Faculty of Medical Sciences, University of Pristina, 38220 Kosovska Mitrovica, Serbia; 5 Institute of Hygiene and Medical Ecology, Faculty of Medicine, University of Belgrade, 11000 Belgrade, Serbia

**Keywords:** Brief Assessment of Cognition in Schizophrenia scale, validation, cross-cultural adaptation, psychometric properties, schizophrenia

## Abstract

The Brief Assessment of Cognition in Schizophrenia (BACS) scale was developed for the assessment of cognitive function in patients with schizophrenia. The objective of this study was to cross-culturally adapt and validate the BACS in to the Serbian language. The study was conducted at the Laza Lazarevic Clinic for Mental Disorders and the Clinic for Psychiatry of the University Clinical Center of Serbia from March 2021 to January 2022. The study enrolled 61 inpatients diagnosed with schizophrenia and 61 healthy controls matched for age and sex. Compared with the healthy control group, the schizophrenia patient group had worse cognitive function in all dimensions measured using BACS (*p* < 0.001 for all measures). The mean standardized composite BACS score was z = −2.46, and symbol coding (z = −2.54) was the most deficient function. Principal component analysis suggests a two-factor structure, where the first factor consisted of loading the measures of verbal and working memory, attention, speed of information processing, and executive function, while the second factor regarded the loading of motor speed. Cronbach’s alpha coefficient demonstrated an excellent level of internal consistency (0.798). These outcomes suggest that the Serbian BACS neurocognitive battery’s psychometric properties are satisfactory, with good overall discriminant validity and high internal consistency. The Serbian BACS appears to be a quick and reliable neuropsychological instrument for evaluating global cognition in schizophrenia patients in Serbia.

## 1. Introduction

Schizophrenia is a severe chronic psychiatric disorder that affects about 1% of the world’s population [1]. It is characterized by psychiatric symptoms such as hallucinations, delusions, and cognitive impairments [2]. Cognitive impairment, as one of the core features of schizophrenia, affects patient function in the real world [2]. Daily living and activities such as work, social problem solving, interpersonal relationships, and overall quality of life are all affected by cognitive impairment [3,4,5]. Consequently, a key imperative to study in schizophrenia research should be the impact of disease on cognitive function and the assessment of the outcomes of therapeutic interventions. Cognitive testing is also one of the best indicators of a patient’s functional and social prognosis in schizophrenia.

In order to identify cognitive dysfunction in schizophrenia patients, numerous neurocognitive batteries have been developed [6], for example, Measurement and Treatment Research to Improve Cognition in Schizophrenia (MATRICS) [7], the Repeatable Battery for the Assessment of Neuropsychological Status (RBANS) [8], and the Cambridge Neuropsychological Test Automated Battery (CANTAB) [9]. However, most are long and complicated, assessing the patient’s entire neuropsychological profile and taking several hours to complete [10].

The Brief Assessment of Cognition in Schizophrenia (BACS) is a reliable and efficient brief performance-based cognition assessment tool that evaluates the major cognitive domains impaired in schizophrenia, including verbal memory, working memory, attention and processing speed, motor speed, verbal fluency, and executive functions [11]. Scores on the BACS are strongly correlated with the real-world function of patients with schizophrenia [12,13] while still being able to reliably delineate the cognitive deficits and functional impairment that patients experience [7,14].

The BACS was created to be easily used by medical professionals such as psychiatrists, psychologists, clinicians, social workers, psychiatric nurses, and other mental health professionals [11]. The test session lasts over 35 min, with just a few minutes left over for scoring compared to more than 2 h for a standard cognitive battery [11]. Researchers evaluating cognitive changes during clinical trials, and clinicians making recommendations on future therapy and drug adaptation could benefit from the availability of a brief and simple tool, such as BACS, for evaluating cognitive function in schizophrenia patients.

The original version of the BACS showed good psychometric properties. High test–retest reliability was found, and the composite BACS score was strongly correlated with the standard battery score [11]. The BACS was translated and validated in more than 30 languages, including English [11], French [15], Brazilian [16], Japanese [17], Italian [18], German [14], Spanish [13], Persian [19], Arabic [20], and Chinese [21]. However, it has not been translated and validated in Serbian. Therefore, the objective of this study was to perform the cross-cultural adaptation and validation of the BACS into the Serbian language and make it available to assist researchers in evaluating schizophrenia-related cognitive impairment and to direct clinical decisions regarding cognitive rehabilitation and interventions for Serbian patients with schizophrenia.

## 2. Materials and Methods

### 2.1. Study Design

This was a cross-sectional study conducted at the Laza Lazarevic Clinic for Mental Disorders and the Clinic for Psychiatry of the University Clinical Center of Serbia from March 2021 to January 2022. The study was carried out in accordance with the Code of Ethics of the World Medical Association, i.e., the Declaration of Helsinki. The study protocol was approved by the Institutional Review Board of the Laza Lazarevic Clinic for Mental Disorders (no: 8371; date: 28 September 2020) and the Ethics Committee of the University Clinical Center of Serbia (no: 623/9; date: 18 September 2020). All of the participants gave their written informed consent, and their anonymity was maintained.

### 2.2. Participants

We recruited 61 inpatients diagnosed with schizophrenia and 61 healthy controls matched for age and sex. Both the patients and the healthy controls were included in the study to examine the discriminant validity of the BACS. The inclusion criteria for patients were: inpatients aged between 18 and 65 years meeting the ICD-10 criteria for schizophrenia, and patients being treated with antipsychotic medications who were in clinical remission. Clinical remission was defined using Remission in Schizophrenia Working Group criteria (i.e., patients with scores 3 or less on items P1, P2, P3, N1, N4, N6, G5 and G9 on Positive and Negative Syndrome Scale). There were no particular prescription requirements that determined who was included in the patient group. The exclusion criteria for the patients were as follows: (1) neurological or neurodegenerative disorders, carcinoma, inflammatory diseases (including acute infection or 3 months after an acute infection), current psychoactive substance use disorder or alcohol abuse, autoimmune diseases, epilepsy, a history of cerebrovascular injury, or head trauma with cognitive sequalae, (2) the use of immunomodulatory or hormonal drugs; (3) intellectual disability; (4) the presence of a neurocognitive disorder of any etiology (confirmed by previous complementary diagnostics) or the suspicion of the presence of a neurocognitive disorder of any etiology (based on clinical assessment methods); (5) currently pregnant or breastfeeding patients. From the medical records, demographic (age, sex, educational level) and clinical (diagnosis, disease duration in years) data of the schizophrenia patients were gathered. The educational level is presented as number of years of education.

The healthy individuals were recruited from the staff members of psychiatric hospitals involved in the research or community members who met the following criteria: age between 18 and 65 years, and the absence of any mental and psychiatric disorders. Exclusion criteria for the healthy controls were same as those for the patients (factors that would influence cognitive performance). Healthy controls were matched for age and sex.

### 2.3. Cross-Cultural Adaptation Procedure

Cross-cultural adaptation and validation of the BACS instrument were made on the basis of internationally accepted principles [22]. The standard forward–backward method was used to translate the BACS from English into Serbian. The original version of the survey was converted into Serbian by two autonomous psychiatrists whose primary language is Serbian. The concepts that the questionnaire was designed to measure were known to one translator, aiming to produce a translation that closely resembled the original, while another was naive and intended to produce a translation that revealed the subtle differences. Discrepancies were resolved by discussion between the translators. This Serbian version was then back-translated into English by two independent native speaking English translators fluent in Serbian, who were blind to the original English versions. Back-translators were unaware of the questionnaire’s concepts in order to avoid bias. Members of an expert committee came to an agreement through consensus after comparing the back-translated version and the original version. The committee included both forward and backward translators as well as methodology experts who were familiar with the construct of interest. No significant issues in both forward and backward translation were encountered. In a pilot study with five schizophrenia patients, the Serbian version of the questionnaire was tested for clarity, comprehension, and acceptability. No significant issues were encountered in the pilot study.

### 2.4. Cognitive Assessment Procedure

The cognitive functions of all patients and healthy controls were assessed using Serbian version of BACS. Psychologist/psychiatrist specialized in the administration of the BACS assessed participants. Five patients and one control did not finish BACS assessment and their data were excluded pairwise. BACS consists of a series of tests that measure the most commonly impaired cognitive aspects that are closely related to the functioning of patients with schizophrenia in the real world. The constructs measured with the Serbian version of the BACS, including explanations of tests, are listed in the order of administration: verbal memory: list learning; working memory: digit sequencing task; motor speed: token motor task; verbal fluency: semantic or category fluency and phonetic or letter fluency; attention and speed of information processing: symbol coding; executive function: Tower of London. After all of the subtests were performed, the composite Serbian BACS score was calculated by comparing the performance of each schizophrenia patient on each subtest with the performance of healthy controls, which was defined as the Z score of the sum.

### 2.5. Statistical Analysis

Quantitative variables were expressed as means and standard deviations or medians with 25th to 75th percentile (according to data distribution), while categorical variables were expressed as absolute frequencies with percentages. The Student’s *t* or Mann–Whitney test was used to assess differences in quantitative variables between patients and healthy controls. For categorical variables, the chi-squared test was used to compare groups. For each subtest of the Serbian version of the BACS, the mean and standard deviation were calculated to obtain standardized (to healthy controls) subtest Z scores on the basis of the following formula: the subtest Z score = (subtest score − subtest mean)/(subtest standard deviation). The composite Serbian BACS scores were calculated using the revised composite BACS score calculation on the basis of sex and age. Pearson’s correlation coefficient was used to assess the intercorrelations between the composite BACS score and its subtests in schizophrenia patients and healthy controls. The structural validity of the Serbian BACS instrument was assessed with principal component analysis. Only items with factor loading ≥ 0.4 were taken into account [23]. Cronbach’s alpha coefficient was used to assess internal consistency of the Serbian version of BACS (alpha range 0–1, where 1 presents perfect reliability). Face validity was assessed using the Student’s *t*-test to compare subtest scores between schizophrenia cases and controls. The threshold for discrimination between schizophrenia patients and controls was determined using the receiver–operator characteristic (ROC) curve, where schizophrenia patients were considered “cases” and controls “noncases”. In addition, sensitivity and specificity, and positive and negative predictive values were calculated. SPSS software version 21 was used for all analyses. A *p*-value of less than 0.05 was considered statistically significant.

## 3. Results

A total of 61 patients with schizophrenia, and 61 age- and sex-matched healthy controls were included in the analysis. The sociodemographic characteristics of the study population are presented in Table 1. While the two groups were well-matched for age and sex, the schizophrenia patient group had significantly fewer years of education than those of the controls. The Serbian BACS version required approximately 40 min for application to the schizophrenia patients and healthy controls, which was similar to BACS versions in other languages.

Table 2 lists the means and standard deviations for all the raw BACS subscale scores for both groups, and the BACS subscale and composite BACS Z scores for patients with schizophrenia standardized to the healthy controls. Compared with the healthy control group, the schizophrenia patient group had worse cognitive function in all dimensions measured using BACS (*p* < 0.001 for all measures).

**Figure 1:**
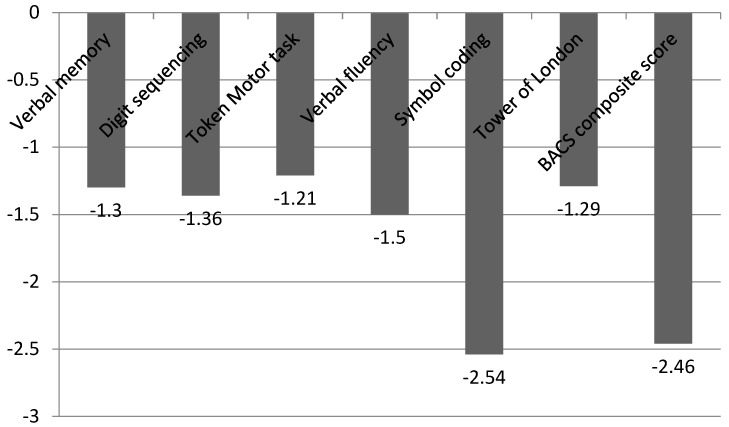
Serbian BACS subtests and composite score in patients with schizophrenia standardized to the healthy controls.

Figure 1 shows the mean scores for the composite BACS score and subtests in patients with schizophrenia standardized to the healthy controls. The mean composite BACS score of the Serbian version was z = −2.46. Symbol coding (z = −2.54) was the most deficient function in Serbian patients with schizophrenia.

The correlations between all BACS measures, and the composite BACS score for schizophrenia patients and healthy controls are presented in Table 3. The patterns of correlations among the groups were very similar. Among patients with schizophrenia, all measures of BACS were strongly correlated with the composite BACS score (*p* < 0.001), justifying the use of the composite score to determine discriminant validity. Among healthy controls, all correlations were significant except the correlation between token motor and the composite BACS score.

Principal component analysis with varimax rotation was used to determine the factor structure of the BACS battery test in the schizophrenia patients’ sample. The factor loadings are shown in Table 4. A two-factor structure was suggested that explained 59.2% of the variance, with the first factor explaining 34.8% of the variance. The first general factor of cognitive function consisted of loading the measures of verbal and working memory, attention, speed of information processing, and executive function, while the second factor regarded the loading of motor speed. The internal consistency of the Serbian version of the BAC was assessed with Cronbach’s alpha, which demonstrated an excellent level of internal consistency (0.798) for the entire scale.

**Figure 2:**
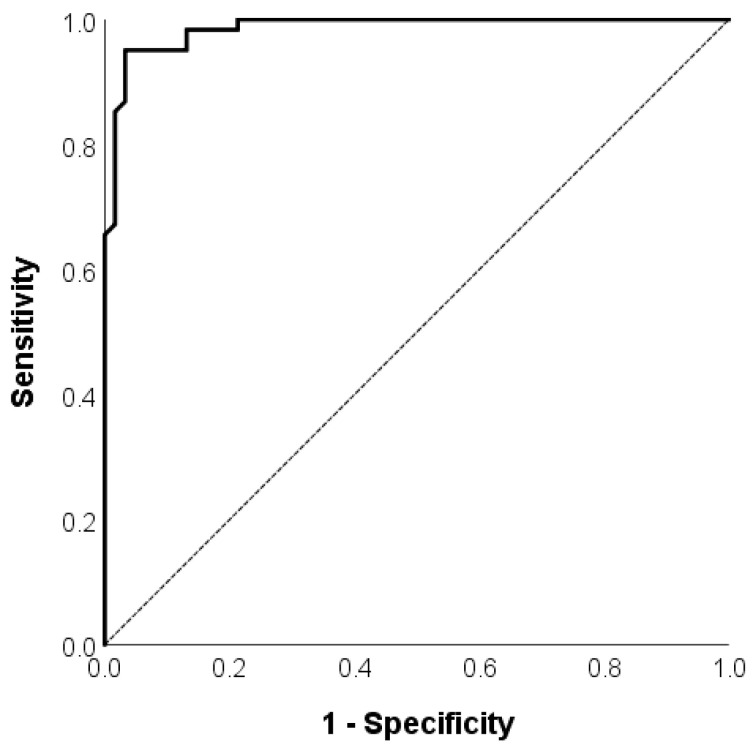
ROC curve of the composite Serbian BACS score for the predicted probability of being identified as a patient or healthy control.

ROC analysis was used to assess the ability of the composite BACS score in distinguishing patients with schizophrenia from healthy controls. The area under the curve for the composite BACS score was 0.986 (confidence interval = 0.971–1.000, *p* < 0.001). The cut-off value for the z score equaled −1.00, the value of the sensitivity was 0.92 and the specificity was 0.97. The findings suggest that the composite Serbian BACS score had good discriminative ability to distinguish between a patient and a healthy control (Figure 2).

## 4. Discussion

The results of our study indicate that the Serbian version of BACS is a suitable instrument to evaluate cognitive function in patients with schizophrenia. The Serbian version of the BACS demonstrated good internal consistency with a two-factor structure, and the capability of distinguish patients with schizophrenia from age- and sex-matched healthy controls.

In our study, on all subtests of the BACS neurocognitive battery and on the composite BACS score, patients with schizophrenia performed significantly worse than the controls. These results are similar to those of the original, English version of BACS and the other language versions of the BACS that demonstrated the discriminant validity of the test. Similar to the original English [11] and a Spanish study [13], the controls in our study did not differ from the patients in terms of age and sex, but had fewer years of education.

In line with the original, English version [11], and the Brazilian [16], German [14], Spanish [13], Persian [19], Arabic [20] and Chinese [21] versions, our findings suggest that the Serbian BACS can be used to distinguish schizophrenia patients from healthy controls on the basis of neurocognitive function. The composite scale and all subscales scores of the Serbian BACS differed significantly between schizophrenia patients and healthy controls. Scores in our sample indicated a higher level of cognitive impairment than that of the sample used to validate the original version [11]. The motor speed task was mostly deficient in our schizophrenia patients’ sample. However, in the original version, verbal memory was listed as the cognitive task with the greatest deficit, followed by attention and processing speed [11]. These discrepancies could be explained by the fact that our patients were hospitalized and might have had more cognitive impairment than that of patients with schizophrenia selected from outpatient clinics. ROC analysis shows that the composite Serbian BACS Z score at the level of −1.00 had high level of sensitivity and specificity to distinguish patients with schizophrenia from controls.

Concerning intercorrelations between the subtests of the BACS and the composite BACS score among patients with schizophrenia, we found large effect correlation (r > 0.50, according to Cohen) [24] for verbal memory, attention, and the speed of information processing and executive function; for working memory and motor speed, there was medium-sized effect correlation (0.30 < r < 0.50). In the healthy controls, there was no correlation between motor speed and the composite BACS score, executive function had a medium-sized effect, while verbal and working memory, attention, and the speed of information processing were in good correlation with the composite BACS score. Lower correlations were found for motor speed tests (r = 0.40) in the Arabic version [22]. The original [11], French [15], German [14], Persian [19], Spanish [13], and Arabic [20] versions all produced results that were comparable. The majority of the intercorrelations in the original English version were significant for schizophrenia patients. The effects were slightly greater in the controls, yet the pattern of associations between the groups was very similar. Our findings confirm that, despite being higher in the control group, the pattern of correlations between each of the subtests and the composite BACS scores was similar across the groups.

A high Cronbach’s alpha, which fell within the acceptable range for internal consistency (0.798), indicates that we achieved a satisfactory level of internal consistency. The high internal consistency of the Serbian version of the BACS is comparable to the analyses of the original English version, and the Japanese, Brazilian, Persian, and Arabic versions [11,16,17,19,20]. According to the results of our study, the Serbian version of the BACS can be used to measure general cognitive function in schizophrenia.

A two-factor structure of the Serbian version of BACS neurocognitive battery was suggested via principal components analysis. A three-factor structure was reported in the original BACS study [11], and the Japanese version [17]. A three-factor structure explained 74.3% of the instrument overall variance in the original version, and 80.3% in the Japanese. In the original version, measures that emphasized motor speed and general cognitive functions were loaded on the first factor, the memory and working memory measures were loaded on the second factor, and executive function was loaded on the third factor. However, in the Serbian two-factor structure, which explained 59.2% of variance, measures that emphasized verbal and working memory, attention, and the speed of information processing and executive function were loaded on the first factor, while the motor speed was loaded on the second factor. The factor analysis in the French validation study also revealed the two-factor solution, which explained 56.6% of the overall variance [15]. Measures that included memory, attention, the speed of information processing, and executive functions were loaded on the first factor, verbal fluency was loaded on the second factor, and motor speed was not associated with this two-factor structure. In contrast, a one-factor solution was obtained in studies of the Spanish, Persian, and Arabic versions [13,19,20]. Even though the proposed number of factors varies, all studies found that a single factor explained most of the variance. The differences in the samples of the various studies may be the cause of this variance in the principal component analysis [9]. The differences in the results between the studies could have been due to differences in sample size or clinical background.

Limitations and future directions. Due to the limited sample size and selection of patients from only two institutions, this study’s findings could not be generalized to the whole population. Therefore, further studies including the assessment of various populations of patients with schizophrenia (treatment of refractory schizophrenia patients and geriatric patients) are needed to fully validate the Serbian version of the BACS. We did not examine testing–retesting or inter-rater reliability. However, the BACS demonstrated good test–retest reliability in previous studies. In the initial BACS development study [11] and in all of the other language versions that had been tested for temporal stability [14,17,19], the global BACS score showed very high correlation between the test and retest sessions. These all confirmed that BACS scores have remained constant over time.

## 5. Conclusions

The Serbian BACS neurocognitive battery’s psychometric properties were satisfactory, with high internal consistency and good overall discriminant validity. These outcomes suggest that the Serbian BACS variant is a valuable instrument for the evaluation of cognitive capability in Serbian-speaking patients with schizophrenia. The BACS appears to be a quick and simple neuropsychological instrument for evaluating global cognition in schizophrenia patients in Serbia.

## Figures and Tables

**Table 1:** Sociodemographic characteristics of patients with schizophrenia and healthy controls.

Sociodemographic Characteristics	Patients with Schizophrenia (*n* = 61)	Healthy Controls (*n* = 61)	*p* Value
Age, years, mean ± SD	36.30 ± 10.31	35.57 ± 10.02	0.696
Sex, n (%)			1.000
Male	33 (54.1%)	33 (54.1%)	
Female	28 (45.9%)	28 (45.9%)	
Education, years, mean ± SD	12.56 ± 1.93	13.52 ± 1.51	0.003
Duration of illness, years, median (25th–75th percentiles)	8 (3–17)		

**Table 2:** BACS battery test performances of patients with schizophrenia and healthy controls.

BACS Battery Test	Patients with Schizophrenia (*n* = 61) Raw Score, Mean ± SD	Z Score of Patients with Schizophrenia	Healthy Control (*n* = 61) Raw Score, Mean ± SD	Raw Score *p*-Value *
Verbal memory	37.28 ± 10.45	−1.30	51.89 ± 8.73	<0.001
Digit sequencing	17.15 ± 3.95	−1.36	21.80 ± 7.15	<0.001
Token Motor task	57.63 ± 10.28	−1.37	79.66 ± 9.43	<0.001
Verbal fluency	38.70 ± 10.58	−1.22	56.33 ± 9.71	<0.001
Symbol coding	33.39 ± 11.67	−2.54	61.16 ± 9.31	<0.001
Tower of London	13.38 ± 3.99	−1.29	17.46 ± 3.08	<0.001
Composite BACS		−2.46		

* *p* values derived from Student’s *t* test.

**Table 3:** Intercorrelations between BACS subtests and the composite score in schizophrenia patients and healthy controls.

	VM	DS	TM	VF	SC	TL	Composite
Composite	0.711 **	0.664 **	0.131	0.591 **	0.585 **	0.426 **	-
TL	0.397 **	0.326 *	−0.197	0.294 *	0.058	-	0.477 **
SC	0.432 **	0.353 **	0.070	0.390 **	-	0.427 **	0.644 **
VF	0.267 *	0.163	0.156	-	0.438 **	0.191	0.576 **
TM	−0.095	0.082	-	0.232	0.405 **	0.232	0.474 **
DS	0.459 **	-	−0.028	0.298 *	0.397 *	0.316 *	0.495 **
VM	-	0.227	0.282*	0.277 *	0.327 *	0.263 *	0.614 **

Correlations in controls above the diagonal; correlations in patients below the diagonal. VM = verbal memory; DS = digit sequencing; TM = token motor task; VF = verbal fluency; SC = symbol coding; TL = Tower of London; Composite = composite BACS score; * *p* < 0.05; ** *p* < 0.01

**Table 4:** Factor loadings of BACS subtests in schizophrenia patients.

	Factor	
	1	2
Verbal memory	0.613	0.142
Digit sequencing	0.609	−0.654
Token Motor task	0.520	0.757
Verbal fluency	0.651	−0.137
Symbol coding	0.820	0.014
Tower of London	0.630	−0.007
Percentage of variances explained	34.8%	59.2%
Cronbach’s alpha	0.920	

## Data Availability

The data presented in this study are available on request from the corresponding author.
